# Trends in time to cancer diagnosis around the period of changing national guidance on referral of symptomatic patients: A serial cross-sectional study using UK electronic healthcare records from 2006–17

**DOI:** 10.1016/j.canep.2020.101805

**Published:** 2020-12

**Authors:** Sarah Price, Anne Spencer, Xiaohui Zhang, Susan Ball, Georgios Lyratzopoulos, Ruben Mujica-Mota, Sal Stapley, Obioha C Ukoumunne, Willie Hamilton

**Affiliations:** a University of Exeter Medical School, Room 1.20 College House, St Luke’s Campus, University of Exeter, Exeter, Devon, EX1 2LU, UK; b Health Economics Group, University of Exeter, Exeter, UK; c University of Exeter Business School, University of Exeter, Exeter, UK; d National Institute for Health Research (NIHR) Applied Research Collaboration (ARC) South West Peninsula, University of Exeter, Exeter, UK; e Institute of Epidemiology and Health Care, University College London, UK; f Academic Unit of Health Economics, University of Leeds, Leeds, UK; g University of Exeter Medical School, University of Exeter, Exeter, UK; h National Institute for Health Research (NIHR) Applied Research Collaboration (ARC) South West Peninsula, University of Exeter, Exeter, UK; i University of Exeter Medical School, University of Exeter, Exeter, UK

**Keywords:** Neoplasms, Early detection of cancer, Clinical decision rules, Health care reform, Primary health care, Diagnostic interval, Time to diagnosis, Semiparametric varying-coefficient model

## Abstract

**Background:**

UK primary-care referral guidance describes the signs, symptoms, and test results (“features”) of undiagnosed cancer. Guidance revision in 2015 liberalised investigation by introducing more low-risk features. We studied adults with cancer whose features were in the 2005 guidance (“Old-NICE”) or were introduced in the revision (“New-NICE”). We compared time to diagnosis between the groups, and its trend over 2006—2017.

**Methods:**

Clinical Practice Research Datalink records were analysed for adults with incident myeloma, breast, bladder, colorectal, lung, oesophageal, ovarian, pancreatic, prostate, stomach or uterine cancers in 1/1/2006–31/12/2017. Cancer-specific features in the year before diagnosis were used to create New-NICE and Old-NICE groups. Diagnostic interval was time between the index feature and diagnosis. Semiparametric varying-coefficient analyses compared diagnostic intervals between New-NICE and Old-NICE groups over 1/1/2006–31/12/2017.

**Results:**

Over all cancers (N = 83,935), median (interquartile range) Old-NICE diagnostic interval rose over 2006–2017, from 51 (20–132) to 64 (30–148) days, with increases in breast (15 vs 25 days), lung (103 vs 135 days), ovarian (65·5 vs 100 days), prostate (80 vs 93 days) and stomach (72·5 vs 102 days) cancers. Median New-NICE values were consistently longer (99, 40–212 in 2006 vs 103, 42–236 days in 2017) than Old-NICE values over all cancers. After guidance revision, New-NICE diagnostic intervals became shorter than Old-NICE values for colorectal cancer.

**Conclusions:**

Despite improvements for colorectal cancer, scope remains to reduce diagnostic intervals for most cancers. Liberalised investigation requires protecting and enhancing cancer-diagnostic services to avoid their becoming a rate-limiting step in the diagnostic pathway.

## Highlights

• Revised UK suspected-cancer guidance liberalised investigation of patients.
• Diagnostic interval was longer for patients with newly introduced referral criteria.
• Scope remains to reduce diagnostic interval further.

## 1 Introduction

Early cancer detection is central to improving outcomes [1]. Most early-detection strategies focus on the timely recognition and investigation of people likely to have undiagnosed cancer [[2], [3], [4]]. As screening detects <6 % of cancer [5], UK strategies focus on promptly recognising the symptoms, signs or test results associated with undiagnosed cancer (“features of possible cancer”, or simply “features”) [6]. In 2005, UK suspected-cancer guidance was published, listing features warranting cancer testing or investigation [7].

The guidance was revised in 2011 for ovarian cancer [8], and in 2015 for remaining cancers [2]. The aim was to expedite cancer diagnosis by lowering the risk of undiagnosed cancer warranting clinical action from ≥5 % to 3 % [2], which was achieved by introducing more vague features into the guidance [2,9]. The revised guidance is officially applicable in England, and endorsed in Wales and Northern Ireland [10].

Our objective was to explore the timeliness of cancer diagnosis in England, Wales and Northern Ireland in 2006–2017 for 11 common internal cancers. We compared time from first feature to diagnosis between two groups: “Old-NICE” (with features of possible cancer in the original 2005 guidance) and “New-NICE” (only participants with features introduced during guidance revision). We hypothesised that times to diagnosis would be longer for New-NICE than for Old-NICE participants, because diagnosing cancer is more challenging and may take longer when symptoms are vague [9,[11], [12], [13], [14]]. We also hypothesised that the difference in time to diagnosis between New-NICE and Old-NICE groups would reduce over time, as evidence on vague cancer features emerged and was translated into practice by guidance revision [2,15].

## 2 Methods

### 2.1 Study setting and design

This serial, cross-sectional, primary-care study used UK Clinical Practice Research Datalink (CPRD GOLD) with linked National Cancer Registration and Analysis Service (NCRAS, Set 15) data. CPRD GOLD comprises prospective, coded, and anonymised medical records from >600 UK general practices, with 389 having NCRAS linkage [16]. The study examined participants in the year before their cancer diagnosis between 2006 and 2017.

### 2.2 Inclusion and exclusion criteria

Inclusion criteria:

• Age ≥18 years
• An incident diagnostic code recorded between 1st January 2006 and 31st December 2017 for **myeloma**(ICD10 C90), **breast**(C50), **bladder**(C67), **colorectal**(C18–C20), **lung**(C34), **oesophageal**(C15), **ovarian**(C56), **pancreatic**(C25), **prostate**(C61), **stomach**(C16), or **uterine**(C54) cancer.
• Practice registration ≥1 year before cancer diagnosis.

These sites were selected because the revised guidance introduced new features of possible cancer for them, allowing participant grouping into “Old-NICE” and “New-NICE” categories (see Section 2.3.3).

Exclusion criteria:

• Scotland, where separate guidance applies [17].
• Multiple primary cancers.
• Cancer typical of the opposite sex; e.g. male breast cancer.
• Screen-detected cancer, identified from NCRAS or by CPRD screening codes in the year before diagnosis.
• No primary care attendance or no recorded feature of the participant’s cancer in the year before diagnosis.

### 2.3 Variables and outcome measures

#### 2.3.1 Features of possible cancer

CPRD codes for features of possible cancer were collated [18], based on the symptoms, signs or blood test results in the original or revised guidance (Table 1) [2,7,8]. Occurrences of these codes, restricted to the relevant cancer site, identified participants presenting with these features in the year before diagnosis. Separate generic “suspected-cancer” codes were identified to explore for changing recording practices.

**Table 1:** Cancer features sought in participants' medical records in the year before diagnosis.

Cancer site	Features listed in NICE 2005 (“Old NICE”)	Features added in NICE 2015 (“New NICE”)
Bladder	Haematuria, visible	Dysuria
	Haematuria, non-visible	Raised white cell count
	Urinary tract infection	
	Abdominal mass	
Breast	Breast lump	Breast pain
	Nipple discharge	Lump in axilla
	Nipple retraction	Other changes of concern, such as distorted breast contour
	Skin changes	
Colorectal	Rectal bleeding	Abdominal pain
	Iron-deficiency anaemia	Faecal occult blood
	Change in bowel habit	Weight loss
	Rectal mass	
	Abdominal mass	
Lung	X-ray findings suggestive of lung cancer	Fatigue
	Haemoptysis	Appetite loss
	Cough	Chest infection
	Dyspnoea	Thrombocytosis
	Chest pain	
	Weight loss	
	Finger clubbing	
	Lymphadenopathy (supraclavicular, cervical)	
	Hoarseness	
	Features suggestive of lung metastases	
	Signs of superior vena cava obstruction	
	Stridor	
	Shoulder pain	
	Chest signs consistent with lung cancer	
Oesophagus and stomach	Dysphagia	Reflux
	Weight loss	Haematemesis
	Upper abdominal pain	Thrombocytosis
	Low haemoglobin/chronic gastrointestinal bleeding	
	Dyspepsia	
	Back pain	
	Upper abdominal mass	
	Suspicious barium meal results	
	Nausea and/or vomiting	
Pancreas	Jaundice	Weight loss
		Diarrhoea
		Back pain
		Abdominal pain
		Nausea and/or vomiting
		Constipation
		New-onset diabetes
Ovary	Abdominal distension/bloating	Early satiety/loss of appetite
	Abdominal pain	Pelvic pain
	Urinary urgency/frequency	Weight loss
	Abdominal/pelvic mass	Fatigue
	Constipation	Change in bowel habit
	Back pain	Raised Ca125
		Ascites
Uterus	Postmenopausal bleeding	High blood glucose
	Abdominal or pelvic mass	Low haemoglobin
	Gynaecological symptoms, such as altered menstrual cycle, intermenstrual bleeding, and post-coital bleeding	Reported haematuria
		Thrombocytosis
	Vaginal discharge	
Prostate	Abnormal digital rectal examination	Erectile dysfunction
	Nocturia	Haematuria, visible
	Urinary frequency	
	Urinary hesitancy	
	Urinary urgency	
	Urinary retention	
	Raised PSA above age-specific value	
Myeloma	Spinal cord compression suspected of being caused by myeloma	Bone pain
	Renal failure suspected of being caused by myeloma	Back pain
		Unexplained fracture
		Hypercalcaemia
		Leukopenia
		Plasma viscosity consistent with myeloma
		Erythrocyte sedimentation rate consistent with myeloma
		Protein electrophoresis suggesting myeloma
		Bence-Jones protein urine test suggesting myeloma

#### 2.3.2 Milestone dates and diagnostic interval

The cancer diagnosis date was the earliest CPRD or NCRAS diagnostic code. The first recorded feature of possible cancer (index feature) was identified, along with the index date. Our outcome variable was “diagnostic interval”: days from index date to diagnosis [19].

#### 2.3.3 NICE grouping

**Fig. 1:**
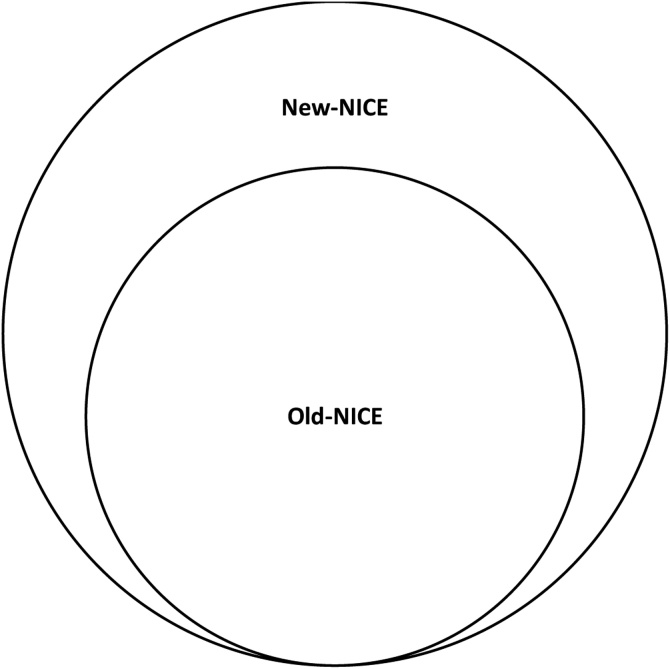
Schematic to show participant grouping. "Old-NICE": participants with a first feature of possible cancer listed in NICE 2005 (including those that have a first feature listed in both NICE 2005 and NICE 2015); “New-NICE”: participants whose first possible feature(s) of cancer is listed solely in NICE 2015 (or in NICE 2011 for ovarian cancer).

Participants were grouped by their index feature(s) (Fig. 1, Table 1):

• Old-NICE: participants with ≥1 index feature from the 2005 guidance [7].
• New-NICE: limited to participants who only had index feature(s) introduced during guidance revision [2,8].

Participants whose only index feature was a generic “suspected-cancer” code were omitted from analyses.

#### 2.3.4 Other variables

Age and sex were identified from the CPRD year of birth, assigning a birthday of 1st July.

### 2.4 Analyses

Simple descriptive statistics summarised age (mean and standard deviation), sex (male, n, %), NICE grouping (New-NICE group, n, %), and the index feature(s) (n, % of all index features). We summarised diagnostic interval using mean (standard deviation) and the 25th, 50th, 75th, and 90th centiles. Diagnostic interval has a skewed distribution and was log-transformed for analyses [13].

Semiparametric varying-coefficient methods estimated coefficients representing the percentage difference in mean log-transformed diagnostic interval between New-NICE and Old-NICE groups (see accompanying methodological paper [20]). A coefficient of 0 represents no difference between the NICE groups. Positive coefficients indicate that diagnostic intervals are longer for the New-NICE than the Old-NICE group; negative coefficients, that they are shorter. The coefficients are estimated on a daily basis, so cannot be reported using a single summary statistic, and are plotted (with 95 % confidence intervals, using bootstrapping, n = 1000 replications [21]) to allow visualisation over 2006—17. The models adjusted for age and sex. Analyses examined each cancer site separately, sample size permitting (package “np” in R) [22].

### 2.5 Study size

For the descriptive statistics, we included all CPRD participants meeting our inclusion criteria. Semiparametric varying-coefficient analyses were limited to cancer sites with participant numbers providing ≥90 % power at the 5 % level to detect a 14-day difference in diagnostic interval between New-NICE and Old-NICE groups. Assuming mean diagnostic intervals of 114 and 100 days, respectively, for the Old-NICE and New-NICE groups, a common standard deviation of 100 days and 10 % of participants classified as New-NICE requires 5980 total participants. An effect size of 14 days matches the two-week-wait target for urgent investigation. We assessed uncertainty in the estimates by confidence interval width.

### 2.6 Missing data and bias

To explore for potential bias associated with changing coding practice, we identified, for annual cohorts: (a) the percentages of participants excluded for having no coded features or only suspected-cancer codes; (b) the proportions of Old-NICE and New-NICE participants; (c) demographic characteristics of participants excluded because they lacked coded features.

## 3 Results

### 3.1 Participants

**Fig. 2:**
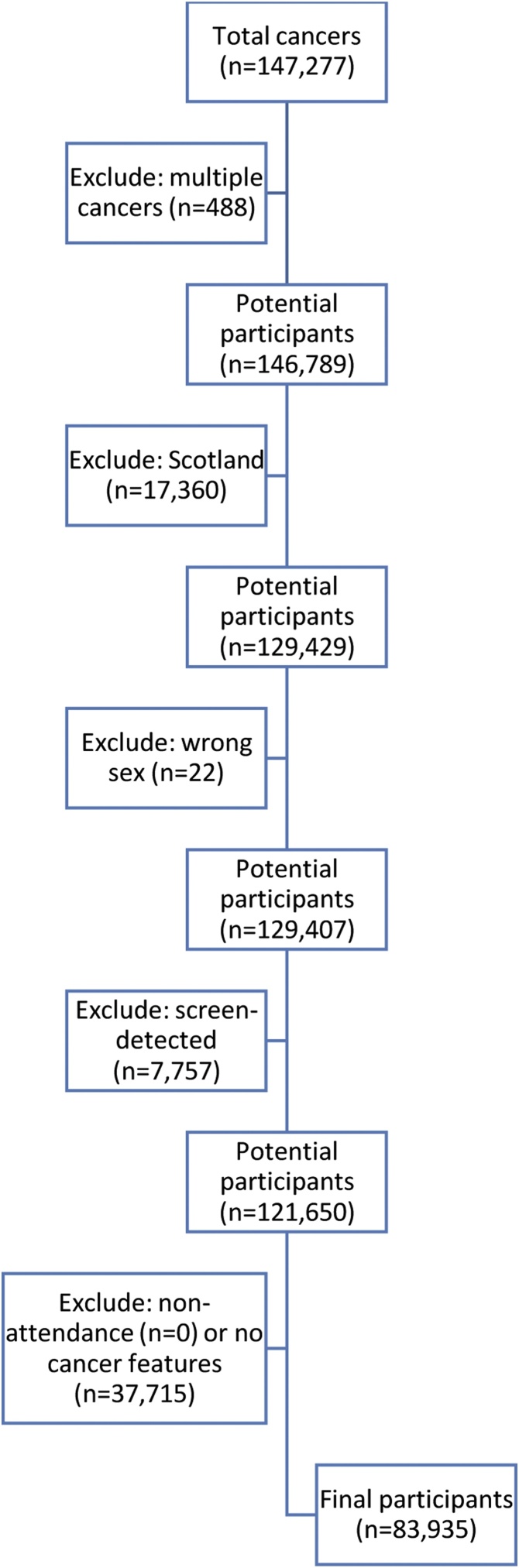
Application of exclusion criteria.

The CPRD provided 147,106 participants, of whom 63,171 (42·9%) were excluded, leaving 83,935 (57·1%) entering the analyses, from 603 practices, of which 384 (63·7%) had NCRAS linkage (Table 2). The main reasons for exclusion were lack of recorded features (n = 37,715), Scottish residence (n = 17,360) and detection following screening (n = 7757) (Fig. 2).

**Table 2:** Numbers of potential inclusions (individual diagnoses), with Cancer Registry linkage, and exclusions, to give final sample sizes by cancer site. The final sample is described in terms of size (N), age (mean, SD), number (%) who are male, and number (%) with an index cancer feature introduced during guidance revision.

Cancer site	Potential inclusions	No. (%) with NCRS linkage	Exclusions	Final sample			
				**N**	**Age, mean (SD)**	**No. (%) male**	**No. (%) in New-NICE group**
Bladder	9030	2583 (28·6)	3787	5243	73·0 (11·5)	3870 (73·8)	799 (15·2)
Breast	37,369	17,452 (46·7)	21,827	15,542	62·9 (16·7)	0 (0)	858 (5·5)
Colorectal	25,011	11,786 (47·1)	13,169	11,842	70·2 (12·6)	6477 (54·7)	5017 (42·4)
Lung	20,033	9080 (45·3)	6926	13,107	71·9 (10·6)	7175 (54·7)	3384 (25·8)
Myeloma	2758	1257 (45·6)	1224	1534	71·0 (11·5)	818 (53·3)	1529 (99·7)
Oesophagus	6041	2710 (44·9)	1769	4272	71·3 (11·8)	2900 (67·9)	451 (10·6)
Ovary	3887	1672 (43·0)	1406	2481	65·5 (13·8)	0 (0)	614 (24·7)
Pancreas	4844	2292 (47·3)	1677	3167	71·7 (11·5)	1580 (49·9)	2672 (84·4)
Prostate	30,083	14,488 (48·2)	8630	21,453	71·6 (9·3)	21,453 (100)	1662 (7·7)
Stomach	3839	1930 (50·3)	1051	2788	73·4 (12·2)	1823 (65·4)	294 (10·5)
Uterus	4382	2124 (48·5)	1876	2506	67·1 (11·3)	0 (0)	713 (28·5)
Total	**147,277**^a^	**67,374 (45**·**7)**	**63,342**^b^	**83,935**	**69**·**6 (12**·**8)**	**46,096 (54**·**9)**	**17,993 (21**·**4)**

a 147,277 cancers in 147,106 participants (of whom 317 had multiple index cancers, including cancer types not in this study).

b 63,342 exclusions in 63,171 patients.

The sex distributions indicate male dominance in **bladder** (3870/5243, 73·8 %), **oesophageal** (2900/4272, 67·9%) and **stomach** (1823/2788, 65·4 %) cancers (Table 2). The overall mean (SD) age at diagnosis (n = 83,935) was 69·6 years (12·8), ranging from 62·9 years (16·7) for **breast** to 73·4 years (12·2) for **stomach** (Table 2).

### 3.2 NICE grouping

The percentage of participants whose index feature was introduced during guidance revision (New-NICE group) varied by cancer, ranging from 1529/1534 (99·7 %) for **myeloma** to 858/15,542 (5·5%) for **breast.** More even distributions were observed for **colorectal** (5017/11,842, 42·4%), **lung** (3384/13,107, 25·8 %), **ovarian** (614/2481, 24·8%), and **uterine** (713/2506, 28·5%) cancers (Table 2).

### 3.3 Index features of cancer

**Breast**, **bladder**, and **prostate** cancers were dominated by one index feature: lump (14,200/15,662, 91·0%), raised prostate-specific antigen (14,473/22,270, 65·0%), and visible haematuria (3435/5346, 64·3%), respectively (Table 3). The remaining sites showed more heterogeneity. **Colorectal** cancer was characterised by abdominal pain (4291/12,084, 35·5%) and rectal bleeding (3913/12,084, 32·4%). For **lung,** cough (4005/13,913, 28·8%), dyspnoea (2876/13,913, 20·7%), and chest infection (2072/13,913, 14·9%) were most frequent. Approximately half of all index features were accounted for by dysphagia (1466/4521, 32·4%) and low haemoglobin (745/4521, 16·5%) in **oesophageal** cancer, and by low haemoglobin (943/3077, 30·6%), upper abdominal pain (479/3077, 15·6%), and dyspepsia (361/3077, 11·7%) in **stomach** cancer. Abdominal pain (925/2669, 34·7%) was most common in **ovarian** cancer, whereas ascites was uncommon (67/2669, 2·5%). **Pancreatic** cancer was characterised by abdominal pain (1068/3259, 32·8%), diabetes (717/3259, 22·0%), and less commonly by jaundice (495/2669, 15·2%). Postmenopausal bleeding accounted for nearly half of all index features of **uterine** cancer (1305/2619, 49·8%), with lower frequencies for high blood glucose (300/2619, 11·5%) and low haemoglobin (275/2619, 10·5%).

**Table 3:** Coded index features of cancer (n, % of total index features presented^a^). Features are listed in order of frequency within cancer site.

Site	Feature	n (% of all index features)
Bladder	Haematuria, visible	3435 (64·5)
	Urinary tract infection	847 (15·9)
	Dysuria	426 (8·0)
	Raised white cell count	427 (8·0)
	Haematuria, non-visible	180 (3·4)
	Abdominal mass	13 (0·2)
	*Total*	5328 *(100)*
Breast	Lump	14,200 (91·0)
	Breast pain	845 (5·4)
	Nipple discharge	253 (1·6)
	Nipple retraction	225 (1·4)
	Other changes of concern	65 (0·4)
	Breast skin changes	44 (0·3)
	Axillary lymph nodes	30 (0·2)
	*Total*	*15,662 (100)*
Colorectal	Abdominal pain	4291 (35·5)
	Rectal bleed	3913 (32·4)
	Change in bowel habit	1940 (16·1)
	Iron-deficiency anaemia	1013 (8·4)
	Weight loss	574 (4·8)
	Abdominal mass	195 (1·6)
	Faecal occult blood	136 (1·1)
	Rectal mass	22 (0·2)
	*Total*	*12,084 (100)*
Lung	Cough	4005 (28·8)
	Dyspnoea	2876 (20·7)
	Chest infection	2072 (14·9)
	Chest pain	1189 (8·5)
	Thrombocytosis	965 (6·9)
	Fatigue	558 (4·0)
	Shoulder pain	520 (3·7)
	Weight loss	485 (3·5)
	Haemoptysis	472 (3·4)
	Signs of lung metastases	270 (1·9)
	Hoarseness	158 (1·1)
	Chest signs consistent with lung cancer	125 (0·9)
	Appetite loss	110 (0·8)
	X-ray findings suggestive of lung cancer	59 (0·4)
	Lymphadenopathy (supraclavicular, cervical)	16 (0·1)
	Finger clubbing	19 (0·1)
	Signs of superior vena cava obstruction	12 (0·1)
	Stridor	2 (0·01)
	*Total*	*13,913 (100)*
Myeloma	Back pain	735 (44·5)
	Abnormal erythrocyte sedimentation rate	426 (25·8)
	Abnormal white cell count	189 (11·5)
	Hypercalcaemia	140 (8·5)
	Plasma viscosity consistent with myeloma	71 (4·3)
	Bone pain	51 (3·1)
	Pathological fracture	11 (0·7)
	Bence Jones protein	11 (0·7)
	Paraprotein	11 (0·7)
	Spinal cord compression suspected of being caused by myeloma	5 (0·3)
	*Total*	1650 *(100)*
Oesophagus	Dysphagia	1466 (32·4)
	Low haemoglobin/chronic gastrointestinal bleeding	745 (16·5)
	Dyspepsia	597 (13·2)
	Upper abdominal pain	402 (8·9)
	Reflux	357 (7·9)
	Back pain	345 (7·6)
	Thrombocytosis	208 (4·6)
	Weight loss	160 (3·5)
	Vomiting	152 (3·4)
	Nausea	61 (1·3)
	Haematemesis	26 (0·6)
	Upper abdominal mass	2 (0·04)
	*Total*	4521 *(100)*
Ovary	Abdominal pain	925 (34·7)
	Raised Ca125	345 (12·9)
	Abdominal distension/bloating	267 (10·0)
	Abdominal/pelvic mass	254 (9·5)
	Back pain	219 (8·2)
	Constipation	201 (7·5)
	Fatigue	132 (4·9)
	Change in bowel habit	83 (3·1)
	Ascites	67 (2·5)
	Pelvic pain	55 (2·1)
	Frequency	53 (2·0)
	Weight loss	48 (1·8)
	Early satiety/appetite loss	14 (0·5)
	Urgency	6 (0·2)
	*Total*	2669 *(100)*
Pancreas	Abdominal pain	1068 (32·8)
	Diabetes	717 (22·0)
	Jaundice	495 (15·2)
	Back pain	373 (11·4)
	Constipation	236 (7·2)
	Weight loss	164 (5·0)
	Nausea	112 (3·4)
	Vomiting	82 (2·5)
	Diarrhoea	12 (0·4)
	*Total*	3259 *(100)*
Prostate	Raised PSA	14,473 (65·0)
	Lower urinary tract symptoms	5649 (25·4)
	Erectile dysfunction	933 (4·2)
	Haematuria, visible	927 (4·2)
	Abnormal digital rectal exam	288 (1·3)
	*Total*	*22,270 (100)*
Stomach`	Low haemoglobin/chronic gastrointestinal bleeding	943 (30·6)
	Upper abdominal pain	479 (15·6)
	Dyspepsia	361 (11·7)
	Dysphagia	260 (8·4)
	Thrombocytosis	241 (7·8)
	Back pain	201 (6·5)
	Reflux	198 (6·4)
	Weight loss	133 (4·3)
	Vomit	130 (4·2)
	Nausea	68 (2·2)
	Haematemesis	57 (1·9)
	Upper abdominal mass	6 (0·2)
	*Total*	3077 *(100)*
Uterus	Postmenopausal bleeding	1305 (49·8)
	High blood glucose	300 (11·5)
	Low haemoglobin	275 (10·5)
	General gynaecological symptoms	247 (9·4)
	Vaginal discharge	218 (8·3)
	Reported haematuria	129 (4·9)
	Thrombocytosis	114 (4·4)
	Abdominal or pelvic mass	31 (1·2)
	*Total*	2619 *(100)*

a Note: Some participants presented with multiple index features; hence, the totals are greater than the final sample sizes.

### 3.4 Diagnostic interval

Overall, the median diagnostic interval was 58 days (interquartile range (IQR) 23–158, N = 83,935). By cancer site, the shortest diagnostic interval was in **breast** (median, IQR: 20, 10–30 days, N = 15,542) and the longest in **lung** (median, IQR: 129, 46–263 days, N = 13,107) (Table 4).

**Table 4:** Diagnostic interval (25th, 50th, 75th, and 90th centiles, mean and standard deviation) by cancer site.

Cancer site	Group	N	Diagnostic interval (days)					
			Centile				Mean	SD
			25^th^	50^th^	75^th^	90^th^		
Bladder	New-NICE	799	61	133	239	322	153·2	106·4
	Old-NICE	4444	32	58	113	226	89·1	84·2
	**Total**	5243	**34**	**64**	**135**	**253**	**98·9**	**90·9**
Breast	New-NICE	858	17	44	138	272	92·4	101·4
	Old-NICE	14,684	10	15	28	53	28·5	43·7
	**Total**	**15,542**	**10**	**16**	**30**	**62**	**32·0**	**50·8**
Colorectal	New-NICE	5017	29	70	159	270	105·4	97·4
	Old-NICE	6825	25	51	105	208	80·7	80·2
	**Total**	**11,842**	**27**	**57**	**126**	**237**	**91·2**	**88·7**
Lung	New-NICE	3384	51	139·5	270	336	160·6	116·9
	Old-NICE	9723	44	124	260	331	152·4	116·9
	**Total**	**13,107**	**46**	**129**	**263**	**332**	**154·5**	**117·0**
Myeloma	New-NICE	1529	37	97	216	307	131·5	109·1
	Old-NICE	5	0·5	4	10	338	70·6	149·5
	**Total**	1534	**37**	**97**	**216**	**307**	**131·3**	**109·2**
Oesophagus	New-NICE	451	38	77	161	280	112·1	96·7
	Old-NICE	3821	21	55	167	293	104·2	106·8
	**Total**	4272	**23**	**57**	**166**	**292**	**105·1**	**105·8**
Ovary	New-NICE	614	26	56	133	281	95·9	99·0
	Old-NICE	1867	34	72	170	283	110·9	100·5
	**Total**	2481	**31**	**67**	**160**	**283**	**107·2**	**100·4**
Pancreas	New-NICE	2672	49	126	258·5	329	154·0	114·6
	Old-NICE	495	11	23	48	91	40·5	53·5
	**Total**	3167	**35**	**97**	**232**	**321**	**136·3**	**115·0**
Prostate	New-NICE	1662	56	123	240	321	151·0	107·9
	Old-NICE	19,791	37	77	174	287	115·1	99·7
	**Total**	**21,453**	**38**	**80**	**181**	**291**	**117·9**	**100·8**
Stomach	New-NICE	294	41	94·5	219	315	133·4	112·0
	Old-NICE	2494	32	88	216	314	127·9	111·7
	**Total**	2788	**33**	**88·5**	**216**	**314**	**128·5**	**111·7**
Uterus	New-NICE	713	76	174	284	337	179·1	112·5
	Old-NICE	1793	25	50	108	206	80·8	80·9
	**Total**	2506	**30**	**67·5**	**167**	**285**	**108·7**	**101·2**
Total	New-NICE	17,993	39	98	222	315	134·2	110·5
	Old-NICE	65,942	21	51	135	271	94·2	99·4
	**Total**	**83,935**	**23**	**58**	**158**	**285**	**102·8**	**103·2**

**Fig. 3:**
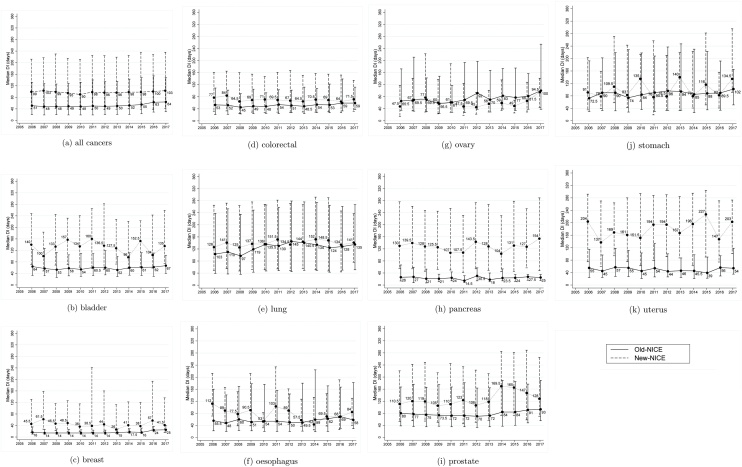
Median (interquartile range) diagnostic interval (days) by year of diagnosis (2006 to 2017), and by NICE grouping: New-NICE (dashed) and Old-NICE (solid).

Median (interquartile range) diagnostic intervals by year and by NICE grouping are plotted in Fig. 3. For all cancers combined, median Old-NICE diagnostic interval was 51 (interquartile range 20–132) days in 2006, compared with 64 (30–148) days in 2017. Median New-NICE diagnostic interval was longer, at 99 (40–212) days in 2006 vs 103 (42–236) days in 2017.

New-NICE diagnostic intervals were considerably and consistently longer than Old-NICE values in **bladder** (133 vs 58 days), **breast** (44 vs 15 days), **pancreatic** (126 vs 23 days), **prostate** (123 vs 77 days), and **uterine** (174 vs 50 days) cancers (Table 4, Fig. 3). Median diagnostic intervals were longer for New-NICE than for Old-NICE participants for **colorectal** (70 vs 51 days), **oesophageal** (77 vs 55 days), and **lung** (139·5 vs 124 days) cancers; however, this difference tended to decrease or disappear over time (Fig. 3). In **ovarian** cancer, diagnostic intervals were shorter in the New-NICE than in the Old-NICE group overall (56 vs 72 days), notably in 2010—16 (Fig. 3).

For **bladder**, **colorectal**, **oesophageal**, **pancreatic** and **uterine** cancers, median Old-NICE diagnostic intervals remained constant over 2006–2017. They were longer in 2017 compared with 2006 for **breast** (25 vs 16 days), **lung** (135 vs 103 days), **ovarian** (100 vs 65·5 days), **prostate** (93 vs 80 days) and **stomach** (102 vs 72·5 days) cancers (Fig. 3).

### 3.5 Semiparametric varying-coefficient analyses

**Fig. 4:**
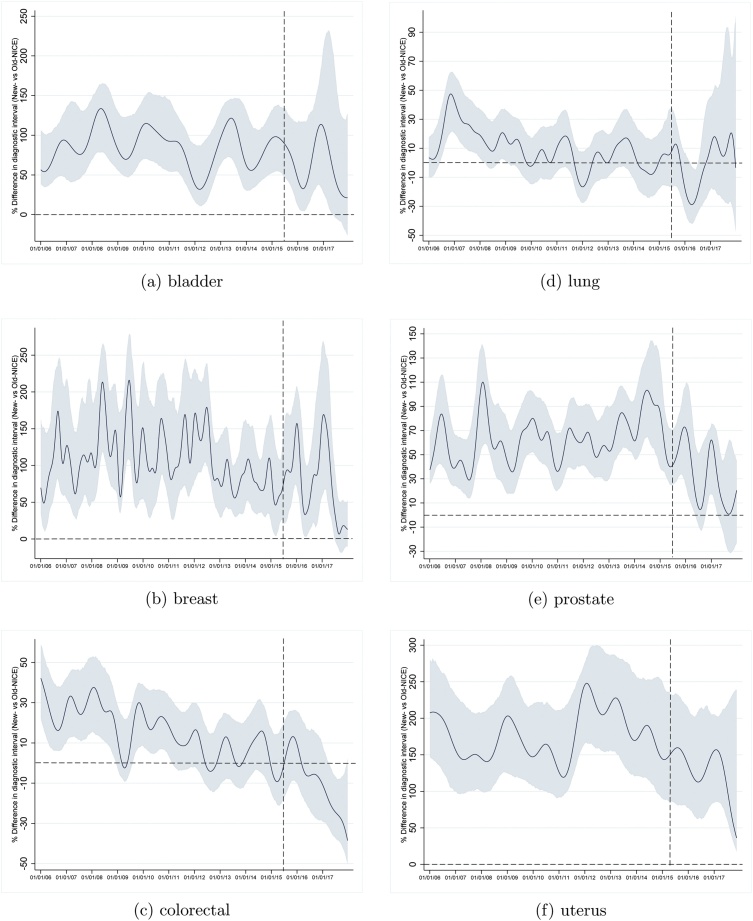
Percentage change in diagnostic interval in New-NICE vs Old-NICE groups, by year of diagnosis (2006 to 2017) for cancers of the bladder, breast, colorectal, lung, prostate, and uterus.

Semiparametric varying-coefficient analyses were powered for **bladder**, **breast**, **colorectal**, **lung**, **prostate** and **uterine** cancers. The percentage differences (with 95 % confidence intervals) in mean log-transformed diagnostic interval between New-NICE and Old-NICE groups over time are plotted in Fig. 4.

After guidance revision on 23rd June 2015, New-NICE diagnostic intervals tended to shorten relative to those of the Old-NICE group in **prostate** (Fig. 4e) and **uterine** (Fig. 4f) cancers (note the downward trajectory towards the horizontal dashed line).

For **colorectal** cancer, the difference in diagnostic interval between the New-NICE and Old-NICE groups reduced over time. After guidance revision, New-NICE diagnostic intervals were shorter than Old-NICE intervals, as indicated by the trend dropping below the horizontal dashed line (Fig. 4c).

For **lung** cancer, New-NICE were longer than Old-NICE diagnostic intervals in the years 2006–10. In 2010–15, there was no difference between the groups. In 2016 (post guidance revision), New-NICE diagnostic intervals shortened relative to Old-NICE diagnostic intervals, but this was not sustained into 2017—18 (Fig. 4d).

### 3.6 Missing data and bias

The proportions of eligible participants excluded for lack of coded features increased over time for **bladder**, **colorectal**, **lung**, **oesophageal**, **ovarian**, **pancreatic**, **stomach,** and **uterine** cancers. This coincided with increased use of suspected-cancer codes (Fig. S1). The demographic details of excluded and included participants were similar (Table S1 and Table 2). The proportions of Old-NICE and New-NICE participants were largely similar across time within cancer sites (Fig. S2).

## 4 Discussion

### 4.1 Findings

This study examined diagnostic intervals for 11 cancers in England, Wales and Northern Ireland over 2006–2017, a period including major revision of national suspected-cancer referral guidance. As hypothesised, times to diagnosis were generally longer for “New-NICE” participants (with index feature(s) of cancer introduced during guidance revision) than for “Old-NICE” participants (with feature(s) in the original guidance). Importantly, for **colorectal** cancer, New-NICE diagnostic intervals were shorter than Old-NICE diagnostic intervals after guidance revision. The gap between New- and Old-NICE groups decreased for **prostate** and **uterine** cancers over time, consistent with decreasing New-NICE diagnostic intervals aided by increasing Old-NICE diagnostic intervals for **prostate** cancer. The revised national guidance and GP responses to its preceding evidence base may have contributed to these changes, along with other early-diagnosis initiatives. In conclusion, scope remains to reduce time to diagnosis for symptomatic cancers in England, Wales and Northern Ireland.

### 4.2 Strengths and limitations

A considerable strength is the study’s primary-care setting, where suspected-cancer guidance is implemented. The CPRD is the largest primary-care database worldwide and is recognised for its high-quality data [23]. We used established methods for case identification [18], with validation of cancer diagnosis by NCRAS where linkage was available. NCRAS data completeness improved in 2013 [24]. Pre-2013 studies report a concordance rate of 83·3% between CPRD and cancer registry information [25]. The CPRD diagnosis date was a median of 11 days (interquartile range –6 to 30 days) later than the registry date pre-2013 for colorectal, lung, gastrointestinal, and urological cancers [26]. Thus pre-2013 diagnostic intervals may be overestimated compared with post-2013 values. Reassuringly, no step-change in New- or Old-NICE diagnostic intervals were observed around 2013, suggesting that any associated bias is small.

We studied diagnostic interval rather than the primary care (time from index date to referral) or secondary care (time from referral to treatment) interval to avoid restricting analyses to participants referred to secondary care [19]. A limitation was the inability to analyse diagnostic intervals separately for participants referred via the two-week-wait pathway [27] because robust data sources for identifying them were unavailable to us.

We found conflicting evidence of changes in GP recording practice over time. The proportion excluded for lack of coded features increased over time for some cancers, often coinciding with increased use of “suspected-cancer” codes. The proportions of Old- and New-NICE groups over time were constant and the similar demographic details for included and excluded participants suggests no marked selection bias. We excluded approximately 26 % of participants for lack of coded features, a proportion consistent with evidence that coded CPRD data identifies 80 % of visible haematuria or jaundice events, and 60–70 % of abdominal pain in patients with pancreatic or bladder cancers [28]. Of participants without recorded features, some will have presented at Emergency Departments without prior primary-care consultations [5,29,30], some will had the information recorded in “free text” [28], and others may have presented with features outside NICE guidance. Such features were deliberately omitted from our study, as irrelevant to our focus on guidance revision.

Our analytical method allowed us to explore trends in the difference in diagnostic interval between groups aligned by their index feature(s) to the revised (New-NICE) or original (Old-NICE) guidance [20]. The method was derived to explore the time-varying and gradual impact of emerging clinical evidence that is legitimised into clinical practice by official guidance revision and implementation [20].

### 4.3 Comparison with existing literature

Our findings build on previous analysis of the original 2005 NICE guideline’s impact on diagnostic interval [13]. Mean diagnostic interval for 15 UK cancers reduced between 2001–2 and 2007–8 by 5·4 days (95 % CI: 2·4–8·5 days) from an initial value of 125·8 days. Similar to our study, median diagnostic intervals were shortest for cancers commonly presenting with lumps/masses (e.g. 26 days for breast) and longest for cancers often presenting with symptoms shared with other diseases (e.g. 112 days in lung cancer) [13]. Our estimates of diagnostic interval for colorectal cancer are similar to those obtained by the International Cancer Benchmarking Partnership using different data sources [31]. Our findings are consistent with the taxonomy of cancer symptom “signatures” and diagnostic difficulty [9]. **Breast** cancer had a narrow signature of a single alarm feature (breast lump) highly predictive of undiagnosed cancer plus the shortest diagnostic interval. In contrast, **lung** cancer had a very broad signature and the longest diagnostic interval.

Jensen et al. [27] investigated the impact of implementing a standardised cancer patient pathway in Denmark in 2007–2009. Post-implementation diagnostic intervals were 15 (12–17) days shorter than peri-implementation values for the 37 % of patients actually referred via a cancer pathway, but were 4 (1–7) days longer for the 63 % of patients diagnosed via other routes. The authors concluded that the cancer pathways expedited diagnosis for a minority of patients.

### 4.4 Clinical interpretation and policy implications of the findings

The relationship between diagnostic interval and mortality (and stage) is U-shaped, reflecting confounding by indication [[32], [33], [34]]. Patients with advanced tumours generally receive an expedited diagnosis (possibly as an emergency) and have poor outcomes because of their high inherent mortality: the so-called “sick-quick”. Conversely, patients presenting with vague symptoms usually have longer diagnostic intervals, and higher mortality – thought to reflect the impact of diagnostic delay, particularly between referral and diagnosis [[32], [33], [34], [35]]. The revised guidance aimed to benefit patients by legitimising doctors to investigate at a lower risk of undiagnosed cancer. This change can reduce both diagnostic delay and emergency presentation. In this study, for colorectal cancer, New-NICE diagnostic intervals reduced relative to Old-NICE interval after guidance revision. This is consistent with general practitioners acting on the vague (“New-NICE”) features introduced during guidance revision. Indeed, the proportion diagnosed via the urgent cancer referral pathway increased from 30 % (95 %CI 29 %–30 %) in 2013 to 33 % (33 %–34 %) in 2016, spanning the period of guidance revision [36].

Our findings of increasing Old-NICE diagnostic intervals over time may reflect growing strain on NHS diagnostic-endoscopy and imaging services [37], as demand for all indications (not just cancer) rises [38], particularly if CT-based targeted screening for lung cancer is introduced [39]. In 2018, inadequate diagnostic capacity was considered a rate-limiting step in the diagnostic pathway [40], and a negative impact of Covid-19 on diagnostic services is already becoming apparent [41].

## 5 Conclusions

We conclude that scope remains to reduce time to cancer diagnosis. The revised colorectal cancer diagnostic guidance may be exerting a downward pressure on time to diagnosis of this cancer, through impacts on the vague features of cancer introduced during guidance revision. Future studies using causal analysis should examine the impact of guidance revision on staging at diagnosis and survival for all cancers, and the possible downstream effects on investigative services. Policy-makers are urged to enhance cancer diagnostic services so that they do not pose a rate-limiting step in the diagnostic pathway, and to protect them from the pressures of Covid-19.

## Funding

This study was funded by 10.13039/501100000289Cancer Research UK [C56843/A21550], who were not involved in any aspect of the conduct of the study, in writing the manuscript or in the decision to submit for publication. This research is also linked to the CanTest Collaborative, which is funded by Cancer Research UK [C8640/A23385], of which WH is co-Director, GL is Associate Director, AS is Senior Faculty, and SP is an affiliated Research Fellow.

SB and OU were supported by the National Institute for Health Research (NIHR)Applied Research Collaboration (ARC) South West Peninsula. The views expressed are those of the author(s) and not necessarily those of the NHS, the NIHR or the Department of Health and Social Care. GL is supported by a Cancer Research UK Advanced Clinician Scientist Fellowship Award [C18081/A18180].

## CRediT authorship contribution statement

**Sarah Price:** Conceptualization, Methodology, Software, Formal analysis, Investigation, Data curation, Writing - original draft, Writing - review & editing, Visualization, Project administration. **Anne Spencer:** Conceptualization, Methodology, Writing - review & editing, Supervision, Project administration, Funding acquisition. **Xiaohui Zhang:** Conceptualization, Methodology, Software, Writing - review & editing, Supervision. **Susan Ball:** Methodology, Writing - review & editing. **Georgios Lyratzopoulos:** Conceptualization, Writing - review & editing, Supervision, Funding acquisition. **Ruben Mujica-Mota:** Conceptualization, Writing - review & editing, Supervision, Funding acquisition. **Sal Stapley:** Conceptualization, Writing - review & editing, Supervision, Funding acquisition. **Obioha C Ukoumunne:** Conceptualization, Writing - review & editing, Supervision, Funding acquisition. **Willie Hamilton:** Conceptualization, Writing - review & editing, Supervision, Funding acquisition.

## Declaration of Competing Interest

WH was clinical lead of the guideline development group which formulated the revised NICE suspected-cancer guidelines (NG12). This paper is written in a personal capacity and is not to be interpreted as representing the views of the Group or of NICE. The remaining authors report no declarations of interest.
