# Supplementary material for: Trends in time to cancer diagnosis around the period of changing national guidance on referral of symptomatic patients: A serial cross-sectional study using UK electronic healthcare records from 2006–17
Source: Cancer Epidemiol. 2020 Dec;69:101805. doi: 10.1016/j.canep.2020.101805 (PMC7480981; doi:10.1016/j.canep.2020.101805)

Figure S1: Percentage of eligible participants excluded because they do not have a coded cancer feature (green) or only have a suspected-cancer code (red) in the eyar before diagnosis. Data are presented by year of diagnosis (2006 to 2017), for bladder, breast, colorectal, lung, myeloma, oesophagus, ovary, pancreas, prostate, stomach and uterus. Only participants with a code for a cancer-specific feature (blue bars) were included in the analyses

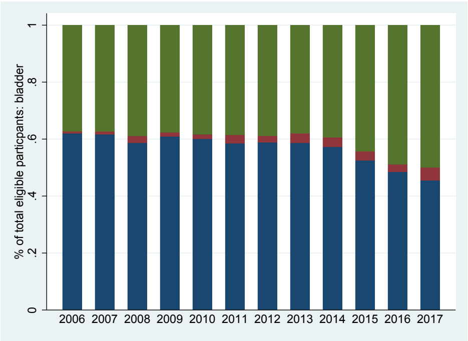

(a) bladder

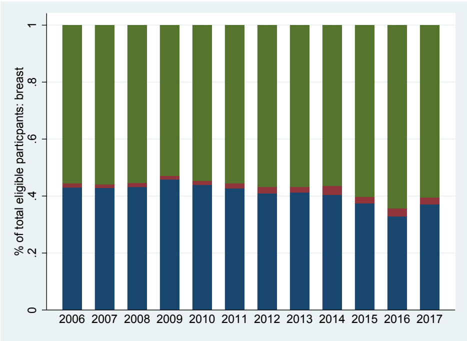

(b) breast

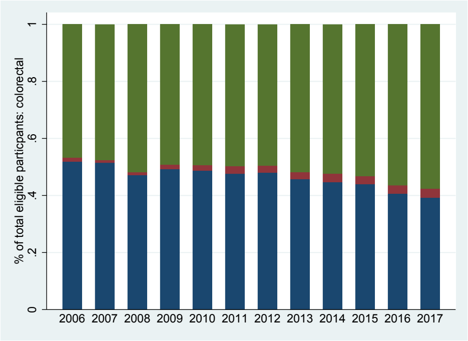

(c) colorectal

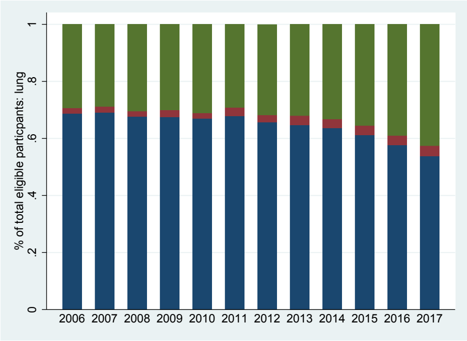

(d) lung

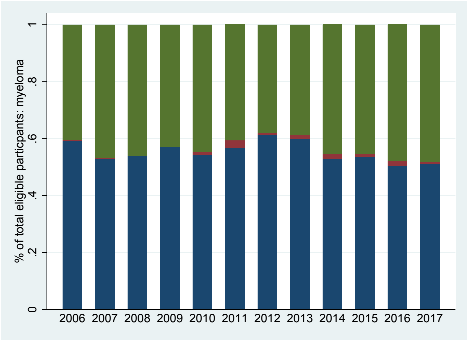

(e) myeloma

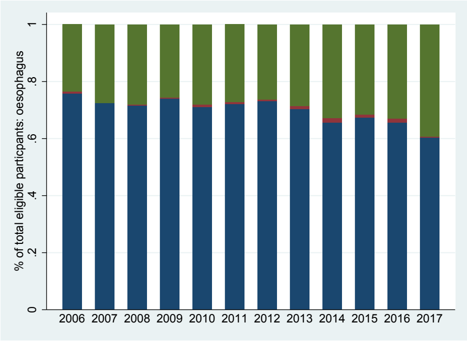

(f) oesophagus

Figure S1: cont.

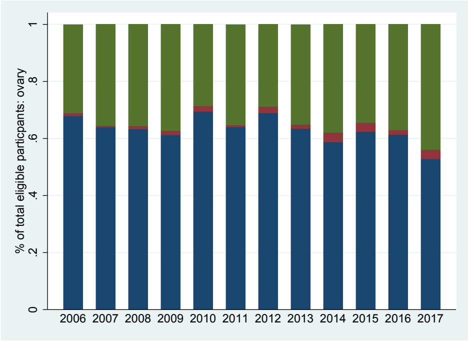

(g) ovary

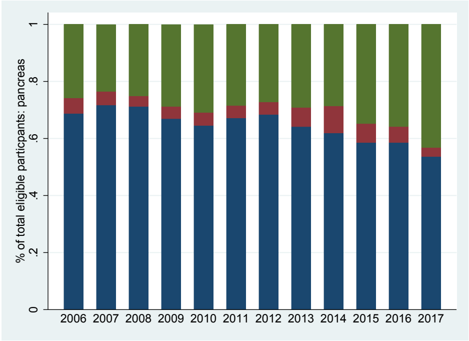

(h) pancreas

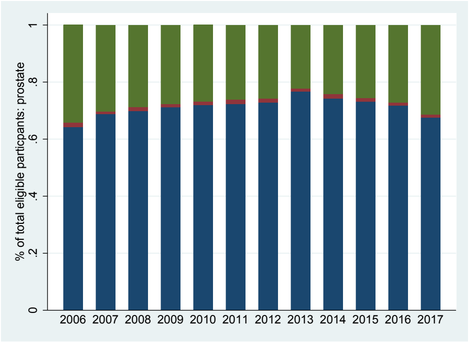

(i) prostate

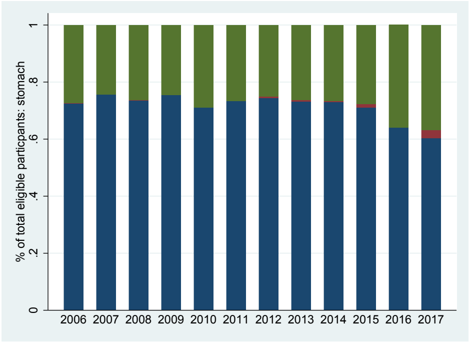

(j) stomach

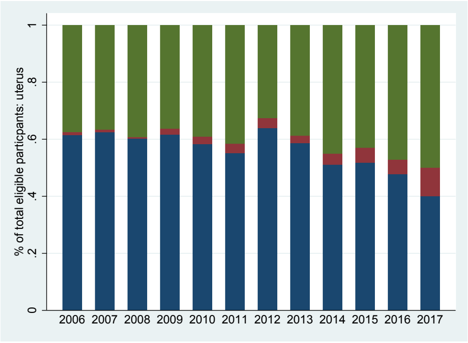

(k) uterus

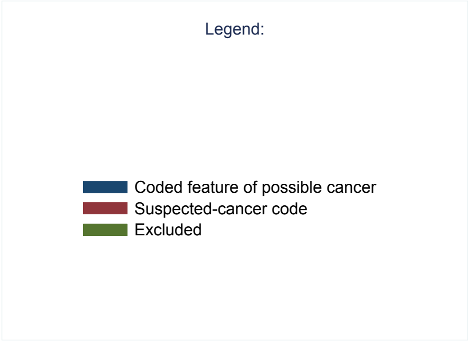

Supplement: Supplementary file 1 [file mmc1.pdf]
